# Response and input time history dataset and numerical models for a miniaturized 3D shear frame under damaged and undamaged conditions

**DOI:** 10.1016/j.dib.2022.108692

**Published:** 2022-10-26

**Authors:** Md Armanul Hoda, Eshwar Kuncham, Subhamoy Sen

**Affiliations:** i4S Laboratory, Indian Institute of Technology Mandi, Mandi, HP, India

**Keywords:** Benchmark, Structural health monitoring, Five-storey steel frame, MATLAB, ABAQUS, Finite element model

## Abstract

A standardized experiment for validating Structural Health Monitoring (SHM) methods is taken up. The test structure is a laboratory-scale five-storey steel frame designed with joints that can be easily detached or reattached as needed. The relatively heavier joints mimic the real-life rigid structural joints fabricated with extensive use of gusset plates and fasteners. The frame members are also proportionally chosen to allow sufficient flexibility as in typical real-life structural frames. The material properties, like elasticity and density are experimentally obtained and reported. The real structure has been tested under different levels of forces exerted through an impact hammer. Accordingly, the force and response histories are recorded and reported in this article.

Further to complement the requirement of support models for typical model based SHM approaches, two support models are prepared that mimic the test setup. The first one is a high-fidelity Finite Element (FE) model prepared using commercial ABAQUS software and the second one is a simplified FE model prepared with MATLAB scripting language. While the first model emphasizes the details to be replicated with sufficient accuracy through the numerical model, the simplified model aims to reduce the computational burden that is typically induced through recursive simulation calls of such support models. Both the models are calibrated (/updated) using typical optimization protocols minimizing the departure between model and real experimental responses. Both time and frequency domain information has been used in this attempt. All details and data produced by the models and the experiments are disseminated in this article.

**Specifications Table**

**Table:** 

Subject	Civil engineering, Structural engineering
Specific subject area	Structural health monitoring, modal analysis, Finite element method, Vibration-based technique, Damage detection, Shear building
Type of data	Table Graph Figure
How the data were acquired	The structure is excited using an impact hammer and structural vibrations are recorded with accelerometers and strain gauge sensors. A data acquisition system and software are used to obtain digital data from the sensors. The software specifications have been detailed in Table 2.
Data format	Raw
Description of data collection	All the laboratory tests reported here have been conducted in the i4S laboratory, Indian Institute of Technology Mandi in India. The operating temperature has been the ambient room temperature which has been observed to be consistent within a range of 15–20 °C. The temperature variation has been considered to be insignificant to cause any substantial thermal induced changes. The test setup is of a three-dimensional frame model that is designed in such a way that its components can easily be assembled or dismantled as needed. Detailed descriptions of all data files used in this paper can be found in the data description section, and the dataset has been uploaded in the Mendeley cloud storage.
Data source location	i4S laboratory, School of engineering, North Campus, Indian Institute of Technology Mandi, Mandi, India.
Data accessibility	Data associated with this article can be found in the Mendeley public repository. Repository name: Mendeley Data Data identification number (DOI): 10.17632/bxmd7c78zf.4 Direct URL to data: https://data.mendeley.com/datasets/bxmd7c78zf

## Value of the Data

• Typical structural health monitoring research attempts to develop a novel methodology to assess structural health. For validation purposes, such studies resort to either numerical experiments or laboratory-scale experiments since damaging a real structure is neither possible nor practical. A benchmarked experiment can however ease the problem wherein the data can be used for validation of any SHM algorithm developed.
• The test structure is a laboratory-scale five-storey steel frame designed with joints that can be easily detached or reattached as needed.
• Detailed experiment on a laboratory frame structure and provide a comprehensive archive of the vibrational response of the test structure containing acceleration, strain, and forcing time histories.
• Further to complement the requirement of support models for typical model based SHM approaches, two support models are prepared that mimic the test setup. The first one is a high-fidelity Finite Element (FE) model prepared using commercial ABAQUS software and the second one is a simplified FE model prepared with MATLAB scripting language.

## 1 Data Description

**Fig. 1:**
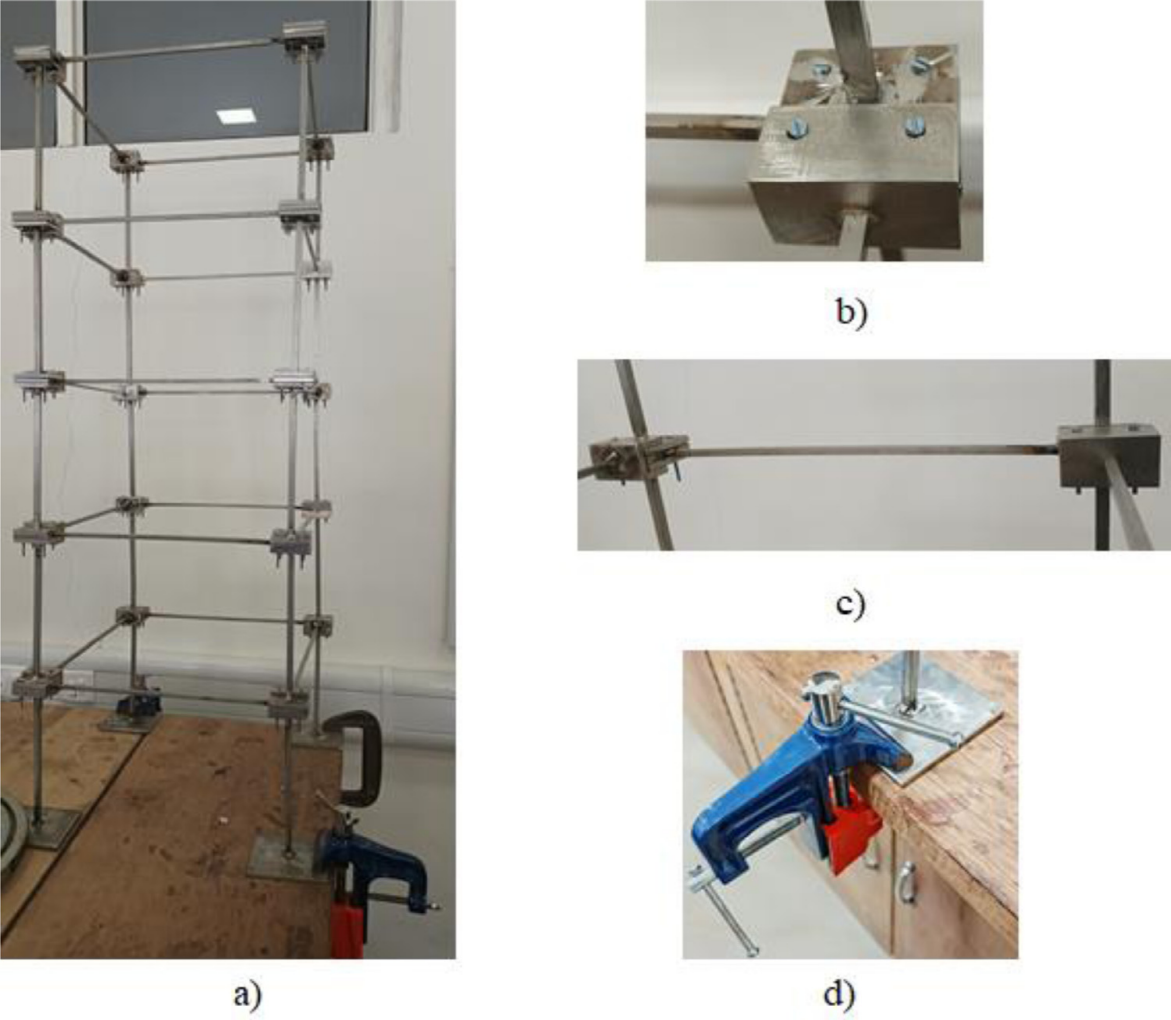
A Laboratory based model of 3D building. (a) Five storey real building, (b) Joint of beam-column, (c) Beams and columns attachments and (d) Base fixed with C-clamp.

**Fig. 2:**
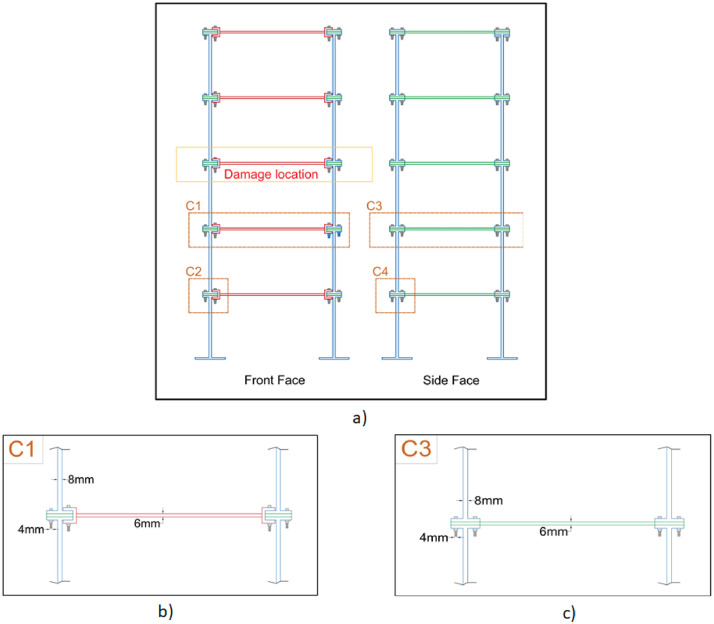
Schematic diagram of the five-storey building frame (a) Elevation and side views of the frame, (b) Beam-column connection (front face), (c) Beam-column connection (side face).

**Fig. 3:**
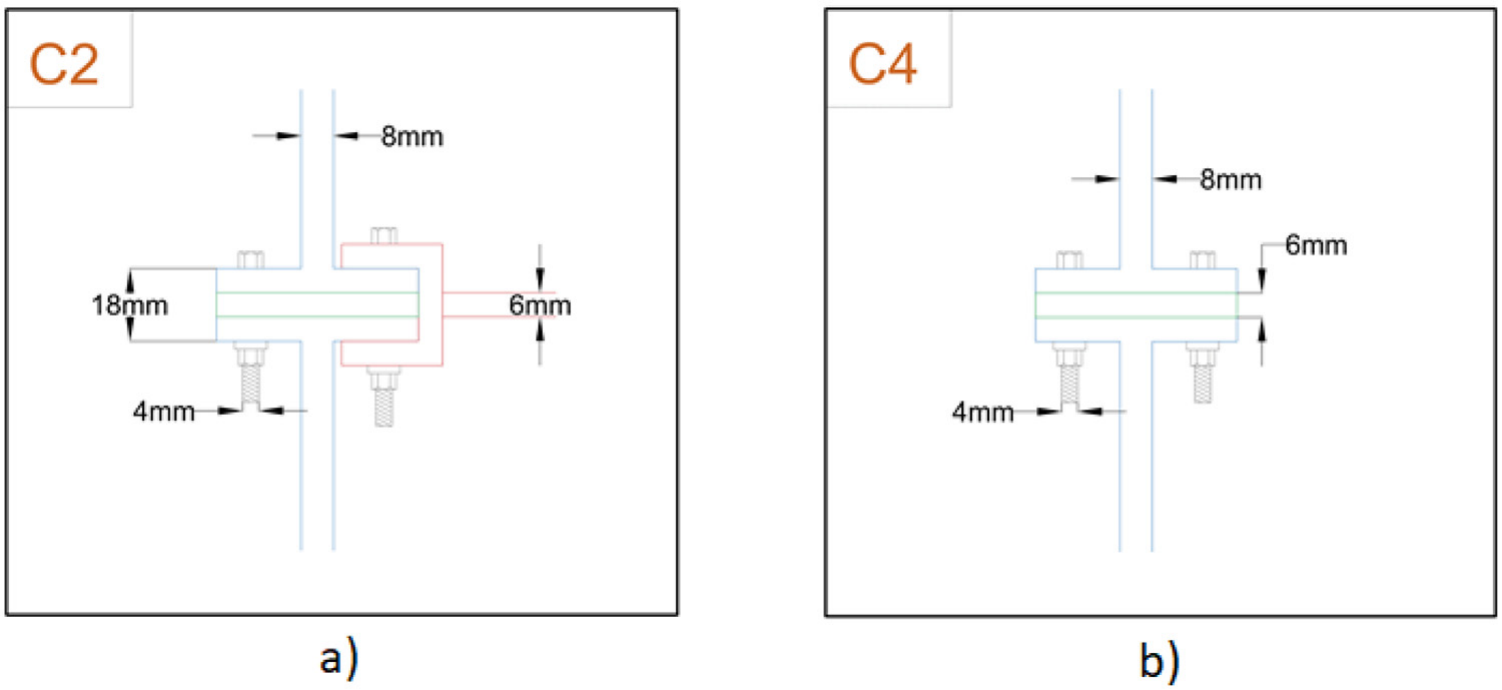
Schematic diagram of beam-column joints (a) Front face (b) Side face.

**Fig. 4:**
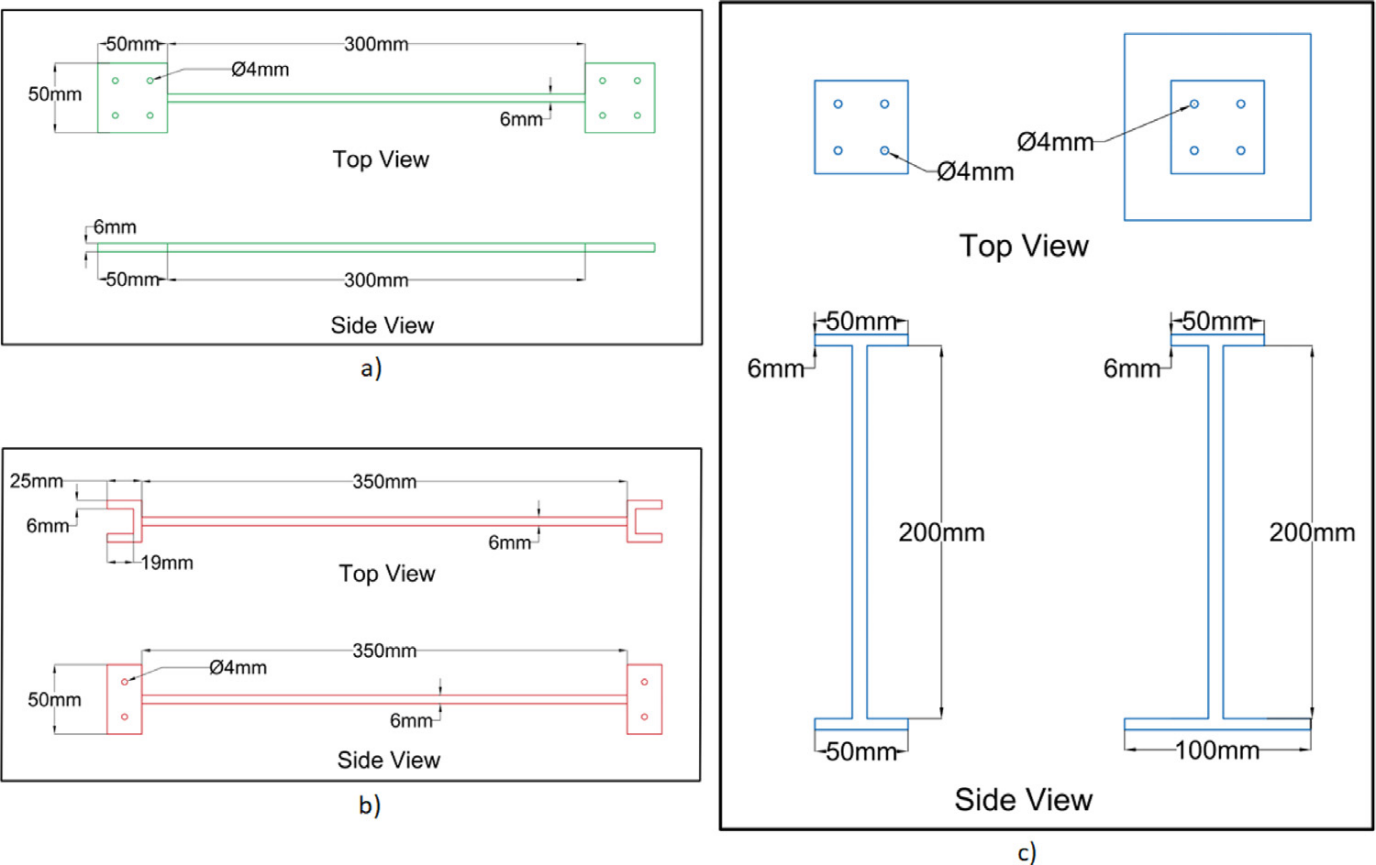
Geometry of a beams and columns (a) Type A beams (b) Type B beams (c) Top and side view of Type X (left) and Type Y (right) columns.

**Fig. 5:**
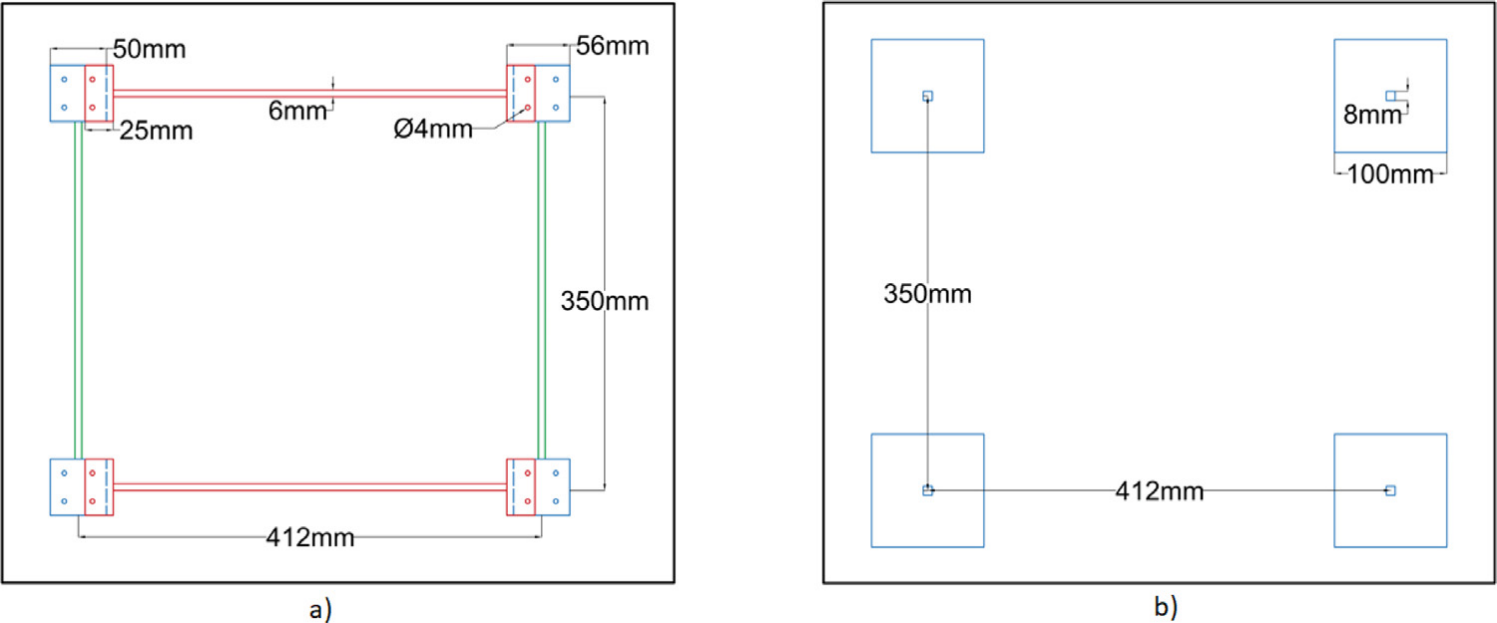
Floor plan view of the frame (a) Top floor plan view, (b) Bottom floor plan view.

**Fig. 6:**
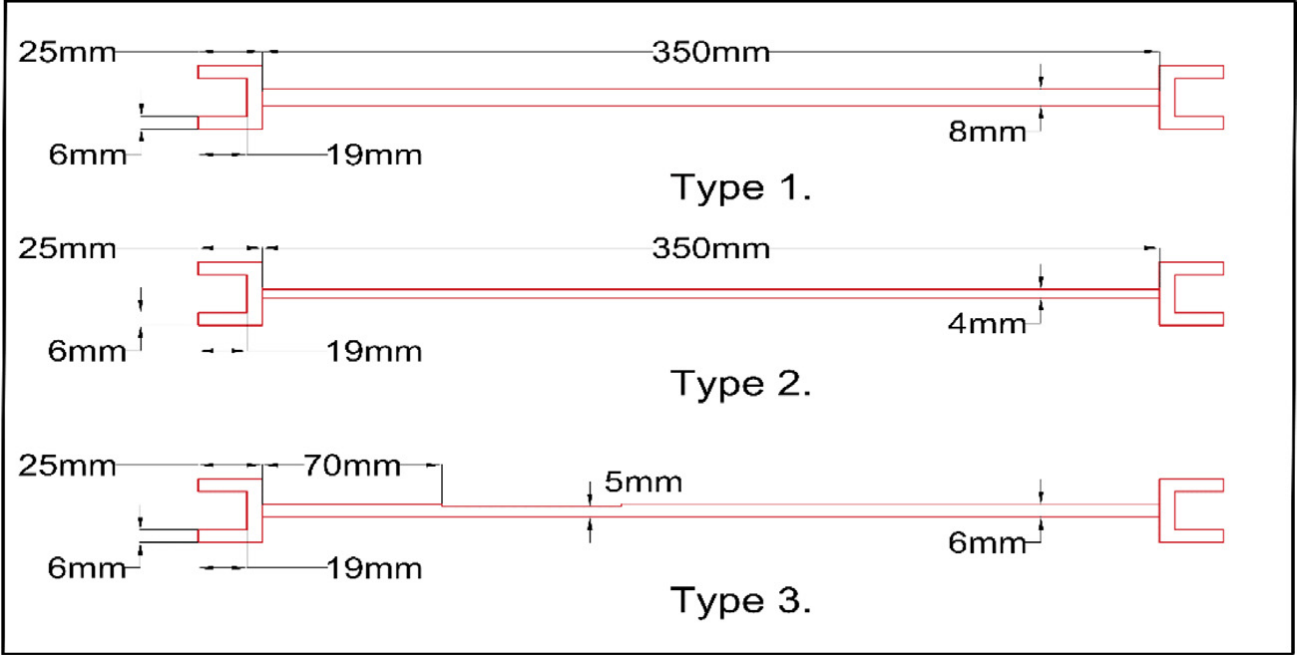
Different types of damaged beam.

A laboratory experiment has been conducted on a five-storey steel frame structure (cf. Fig. 1, Fig. 2, Fig. 3, Fig. 4, Fig. 5 and 8, Table 1) under impact load and vibration response such as acceleration, strain, and excitation force (cf. Fig. 11) are sampled at 500 Hz for damaged and undamaged cases. The damage is induced by replacing a healthy beam with a damaged one (cf. Figs. 2a and 6) in the third storey of the frame. Force is recorded using an impact hammer, and acceleration and strain data are recorded using piezoelectric accelerometers and strain sensors (cf. Fig. 10, for specification see Table 2). The sensor data is fetched with a high-end data acquisition system and further recorded with the help of an interpreter software. A modal frequency response function (FRF) or frequency domain decomposition (FDD) can be used to extract the modal parameters of a structure using the sensor data [1,9].

**Table 1:** Physical parameter of stainless steel 304 grade.

Modulus of Elasticity (GPa)	Shear modulus (GPa)	Density (kg/m^3^)	Poisson's ratio
188.5	75.5	8067	0.265

**Table 2:** Detailed specification of Instruments.

Instruments	Company Name	Specifications
Accelerometer TE connectivity	Accelerometer TE connectivity	Model Number: 7104A-0050/7101A-0050 Accelerometer Type: IEPE Measurement Range: 50 g Frequency Range: 0.3/0.5–10,000 Hz Sensitivity: 100 mV/g Co-variance (g2): 7.73 × 10^−8^ Operating Temperature: -55 to 125 °C Weight: 8.6/4 g
Strain Sensor PCB	Piezotronics	Model Number: 740B02 Strain Sensor Type: ICP Measurement Range: 100 pk μ∈ Frequency Range: 0.5–100,000 Hz Sensitivity (*pm* 20%): 50 mV/μ∈ Transverse Sensitivity ≤ 5% Co-variance ((μ∈) 2): 3.30 × 10^−5^ Overload Limit (Shock): *pm*10,000 g p Operating Temperature: -53 to 121 °C Acceleration Sensitivity: 0.001 μ∈/g Weight: 0.5 g
Impact Hammer	PCB Piezotronics	Model Number: 086C03 Hammer Type: ICP Measurement Range: ±2224 N pk Resonant Frequency: ≥22 kHz Sensitivity (*pm*15%): 2.25 mV/N Co-variance (N2): 2.50 × 10^−5^
DAQ System	KRYPTON	Number of channels: 8 (8 × ACC), 4 (4 × ACC), 4 (3 × strain, hammer), Sampling Rate: 20 kS/s per channel Voltage Ranges: ±10 V Typical Dynamic Range @10 kS: -140 dB Typical noise floor @10 kS: -109, 43 dB Operating Temperature: -40 to 85 °C
Software	DewesoftX	Measures the sensor response in time domain

Further, typical model based SHM approaches may demand a support model for the structure to be investigated [2,3]. For most of the benchmarked experiments, this model is typically not being supplied with. Eventually, to make use of the benchmark data, the investigator must prepare her/his own model of the test set up without being sure if at all the model is replicating the reality with sufficient accuracy or not. This might lead to confusion regarding the potential of the developed algorithm. This issue is addressed through supporting the response archive with two numerical models that mimic the reality with sufficient accuracy. Two models are prepared in this attempt: 1. A high fidelity ABAQUS-based finite element model (cf. Fig. 12) and 2. A wireframe model prepared using MATLAB (cf. Fig. 14). The second one is intended for the algorithms requiring recursive simulation of the support model [4]. Nevertheless, it is always expected that no amount of effort in modeling can exactly mimic the reality. Yet, a primary model that approximately matches the structure's modal and time-domain responses, can safely be considered in any model-based SHM approach while the departure can be handled within the model uncertainty.

Further, few example cases of damages in both the real structure and its numerical replica are performed with frequencies comparison (Table 3, Table 4, Table 5). The resulting data can be employed for validating damage detection approaches [3,5,6]. All details pertaining to the real experiment and modeling have been vividly demonstrated in the following.

**Table 3:** Comparison of natural frequencies (Hz) for undamaged section

			Deviation (%)		
Experimental	ABAQUS	MATLAB	∈ae	∈me	∈ma
5.524	6.254	6.227	13.206	12.719	0.430
6.775	6.577	6.534	2.923	3.556	0.652
-	7.893	7.962	-	-	0.874
20.390	21.428	21.295	5.091	4.438	0.621
23.070	22.286	22.117	3.398	4.131	0.759
-	24.198	23.959	-	-	0.989

**Table 4:** Comparison of natural frequencies (Hz) for type 1 damage.

			Deviation (%)		
Experimental	ABAQUS	MATLAB	∈ae	∈me	∈ma
5.585	6.561	6.517	17.481	5.585	6.561
6.866	6.565	6.534	4.391	6.866	6.565
-	8.057	8.120	-	-	8.057
20.360	21.913	21.800	7.628	20.360	21.913
22.830	22.232	22.056	2.619	22.830	22.232
-	24.837	24.631	-	-	24.837

**Table 5:** Comparison of natural frequencies (Hz) for type 2 damage.

			Deviation (%)		
Experimental	ABAQUS	MATLAB	∈ae	∈me	∈ma
5.493	5.935	5.909	8.050	5.493	5.935
6.744	6.593	6.546	2.245	6.744	6.593
-	7.801	7.866	-	-	7.801
19.560	21.003	20.835	7.377	19.560	21.003
22.890	22.346	22.161	2.377	22.890	22.346
-	23.417	23.133	-	-	23.417

All the laboratory tests reported here have been conducted in the i4S laboratory, Indian Institute of Technology Mandi in India. The operating temperature has been the ambient room temperature which has been observed to be consistent within a range of 15–20 °C which has been considered insignificant to cause any substantial thermal induced changes. The test setup is of a three-dimensional frame model that is designed in such a convenient manner that elements of this frame can be easily assembled or dismantled as needed without much complexity.

The frame is excited using an impact hammer in different directions and locations (for more details see the Section 2.4). A total of sixteen datasets for undamaged and three damaged conditions of the structure are recorded and shared in the raw format.

Data contains following folders as-*Experimental Data***:** A undamaged_section folder which contains Data file name as und_position1_28th_jan.xlsx with the abbreviations should be read as: und- undamaged, position1 –force is applied at 1st position, 28th_jan – 28th January 2022, similarly for damage types (Folder named as e.g., Damaged_type1) are represented as heavy, lightbeam, reduc for type 1, 2 and 3 damages, respectively. Supporting MATLAB codes are also attached in respective folders. *MATLAB Model***:** This folder contains 3 excel file as Damaged_type1, Damaged_type2, Undamaged, which represent heavy, light beam and undamaged beam, respectively. These folders contain MATLAB files of 3D frame structure. *ABAQUS Model:* This folder contains 4 folders named as Damaged_type1, Damaged_type2, Damaged_type3, Undamaged, which represent heavy, light beam, reduced cross section beam and undamaged beam, respectively. The folder contains ABAQUS files (e.g., project1.cae, project1.jnl) of 3D frame structure. *Figures used***:** In this folder all figures are provided which is used in this paper. *Dynamic Analysis***:** This folder contains Abaqus1.xlsx file in which the acceleration responses obtained through Abaqus as well as experiments are stored. Within MATLAB file (Acceleration_comparision.m) both these responses are compared. *Frequencies comparison:* The comparision.xlsx file within this folder, compares frequencies of undamaged and all three damaged conditions with respect to the real as well as numerical (simulated) data. *Nano-indentation test Data***:** This data is used for the determination of modulus of elasticity of material. In this folder, there are three files named as sample_pos (1,2,3) corresponding to three different positions of the sample. *Noise Data***:** Within this folder, main.m file demonstrates the noise and covariance of noise of the sensors used (accelerometer, strain sensor, impact hammer) and an excel file named Test_cov.xlsx stores the noise of respective sensors. *Nonlinear Stress-Strain data***:** This folder contains 2 file (rambur_osgoods.m and ramburg osgoods.xlsx) which contains actual stress-strain data and the data optimized through Ramburg-Osgoods assumption [4].

## 2 Experimental Design, Materials and Methods

All the laboratory tests reported here have been conducted in the i4S laboratory, Indian Institute of Technology Mandi in India. The operating temperature has been the ambient room temperature which has been observed to be consistent within a range of 15–20 ^o^C. The other details related to material, geometry, and experimental procedure are meticulously presented in the following.

### 2.1 Geometric Details of the Frame

The test setup is of a three-dimensional frame model (cf. Fig. 1a) that is designed in such a convenient manner that elements of this frame can be easily assembled or dismantled as needed without much complexity (cf. Fig. 1b). The entire structure is composed of 8 mm x 8 mm column and 6 mm x 6 mm beam elements (twenty numbers each) assembled to realize the 3D structural configuration with the help of 40 joints. The ends of the beams and columns are attached with a C-shaped joint arrangement that makes the assembly easily configurable (cf. Fig. 1c).

The structure is held in its place through a specially fabricated column set with its ends being welded to a 6mm thick plate (cf. Fig. 1d). Finally, the ends of the bottom columns are further fastened to a wooden testbed with the help of a set of C-type bench vise clamps [7].

The schematic diagram of the whole frame is shown in Fig. 2a with its front and side face along with the beam connections detailed in Fig. 2b and c. Alongside, the schematic diagrams for beam-column joints of front as well as side-faces have been detailed in Fig. 3a and b.

The distance between the end arrangement is denoted in this article as clear length. Depending on the length, the beams are categorized into two groups: Type A of clear length 300 mm (node to node distance of 350 mm) and Type B of clear length 350 mm (node to node distance of 412 mm). The mentioned beam types (i.e., Type A and B) are further detailed in Fig. 4a (Type A) and Fig. 4b (Type B), respectively.

All the columns are fabricated with a clear length of 200 mm uniformly. Due to the requirement of base fixity, the bottom columns (denoted here as Type X) are fabricated with larger base plates (100 mm x 100 mm) compared to other column bases (denoted as Type Y with 50 mm x 50 mm) as detailed in Fig. 4c. Accordingly, the bottom floor plan is slightly different from the other floor plans. Both types of floor plans are further detailed in Fig. 5 [4,7].

The type B beams were subjected to three types of structural anomalies. Type 1- wherein the cross-section of a beam element has been increased to 8 mm x 8 mm, Type 2- wherein the cross-section has been reduced to 4 mm x 4 mm and finally, Type 3 - wherein the beam has been damaged partially by reducing its cross-section by 1mm in width and depth in its second of five equal segments. The details of this damage can be found in Fig. 6.

### 2.2 Material Property Estimation

Each element is made with stainless steel of 304 grade. Even though typically, such materials are supplied with a list of the material specification, it is always suggested to run a cross-check for the physical properties. The properties reported in Table 1 are obtained through performing real experiments on the material, e.g., modulus of Elasticity was further cross-checked through nano-indentation test, while the density has been obtained manually (Mass/Volume), and Poisson's ratio has been directly taken from a material testing site [8], etc. The nonlinear property of the material is obtained through fitting a Ramberg-Osgood model to its physical stress-strain data which has been detailed in the following.

### 2.3 Ramberg Osgood's Relationship/Model

**Fig. 7:**
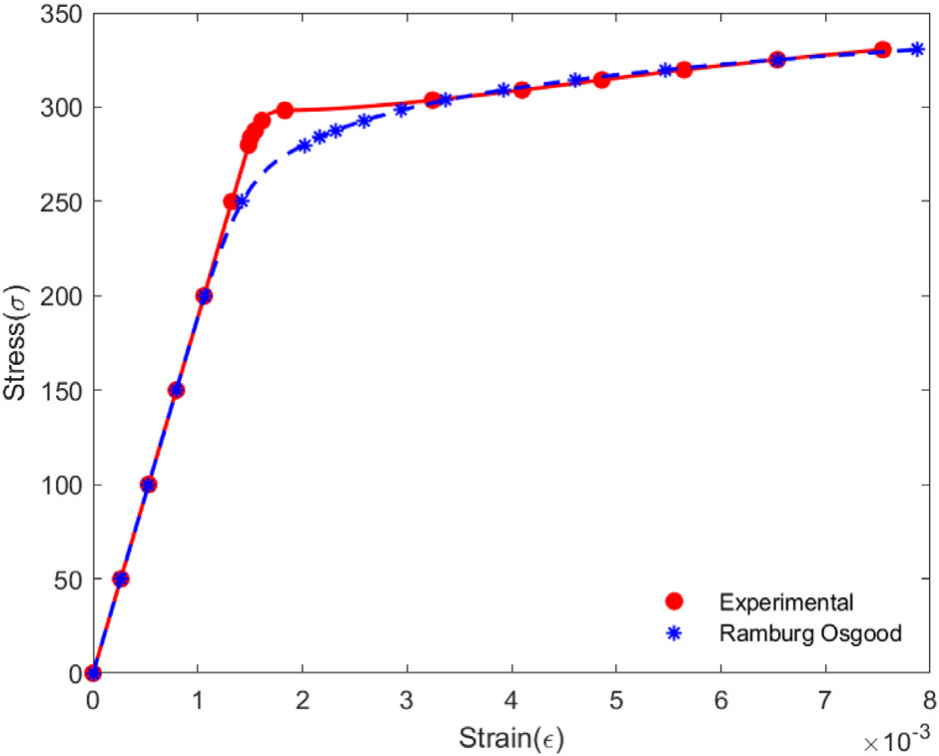
Stress-strain curve fitted with Ramberg-Osgood model.

The Ramberg-Osgood model establishes the nonlinear stress-strain relationship and is generally suggested especially for metals to identify the pertinent strain hardening regions more accurately. This model provides a smooth transition from elastic to plastic regions. The Ramberg-Osgood model can be defined using the following equation,

$$\varepsilon =\frac{\sigma }{E}+\alpha \frac{\sigma }{E}{\left(\frac{\sigma }{\sigma o}\right)}^{\eta_{-1}}$$

where σ denotes stress at any location and ε is the associated strain. E is the modulus of elasticity, and yield stress is represented by σo . α and η are Ramberg-Osgood's parameters. Exploiting this relationship, the strain at the stress levels can be calculated with an initial guess for α and η .Finally, one needs to run an elaborate optimization protocol to have a set of model parameters (i.e., α and η) through minimizing the departure between the physical stress-strain curve to that obtained through the Ramberg-Osgood model. Generalized Reduced Gradient optimization technique has been employed for this optimization and the final stress-strain curve following the Ramberg-Osgood model with optimized model parameters is presented in Fig. 7.

### 2.4 Instrumentation Details

**Fig. 8:**
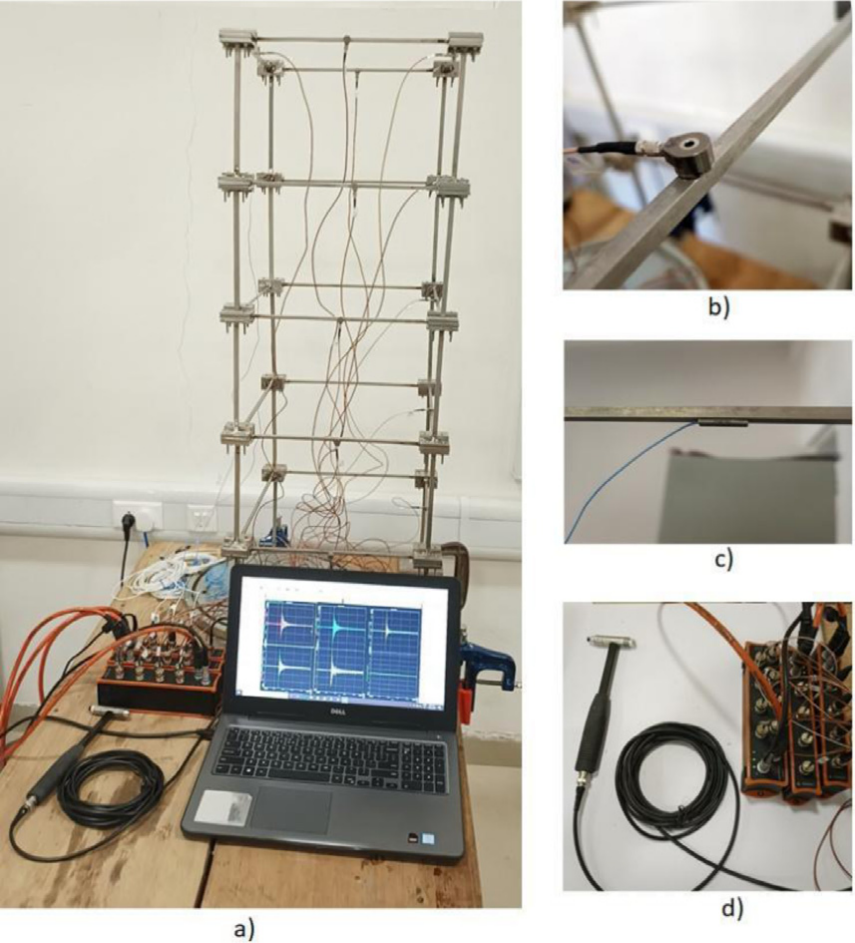
Experimental setup (a) Assembled frame with attached instrument (b) Accelerometer (c) Strain sensor (d) Impact hammer and DAQ.

**Fig. 9:**
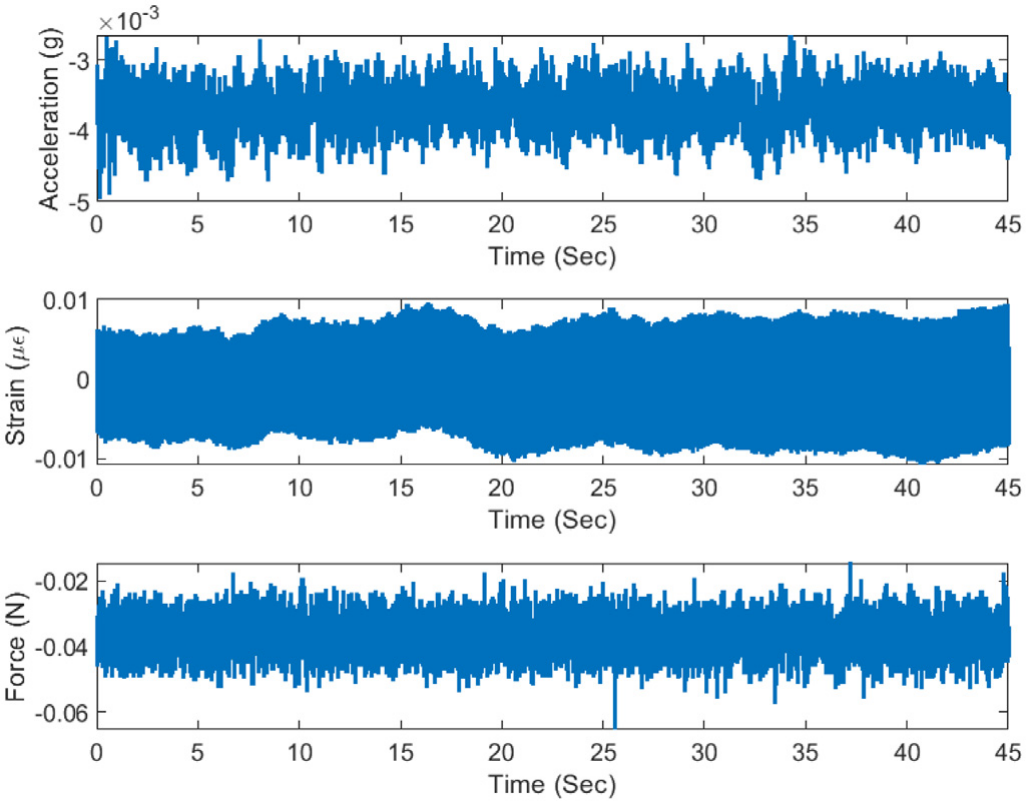
Measured noise response.

The frame is excited using an impact hammer fitted with a piezo-electric tip from which the forcing history can be retrieved. A total of twelve uni-axial ICP (Integrated circuit Piezoelectric) type accelerometers (ACC) (cf. Fig. 8b) are attached at the different locations of the structure (detailed later) and sampled using a Data Acquisition (DAQ) system at constant sampling frequencies of 500 Hz. A set of three ICP type strain gauge sensors (cf. Fig. 8c) are also attached to the structure at various locations to sample dynamic strain response at the same sampling frequency as with the accelerometers. The location and orientation of the excitation, as well as the sensing, are altered regularly. For each case scenario (damaged or undamaged), three sets of time series (Three subsequent hammer hits are ensured approximately within 30 s which are attempted to be approximately equidistant in time) are recorded under the same forcing, instrumentation, and health conditions. Sample figures of sensor attachments (cf. Fig. 8a) and impact hammer are presented in Fig. 8d. The noise (due to electrical noise) data of each instrument has been measured by connecting the DAQ with the wire while the sensor is fitted loosely to it, incapable of transferring vibration responses. Typically, this is a case of sensor fault wherein sensor noise only gets registered [10] (cf. Fig. 9) and co-variance has been taken out (reported in Table 2). The detailed specifications for each instrument are listed in Table 2.

**Fig. 10:**
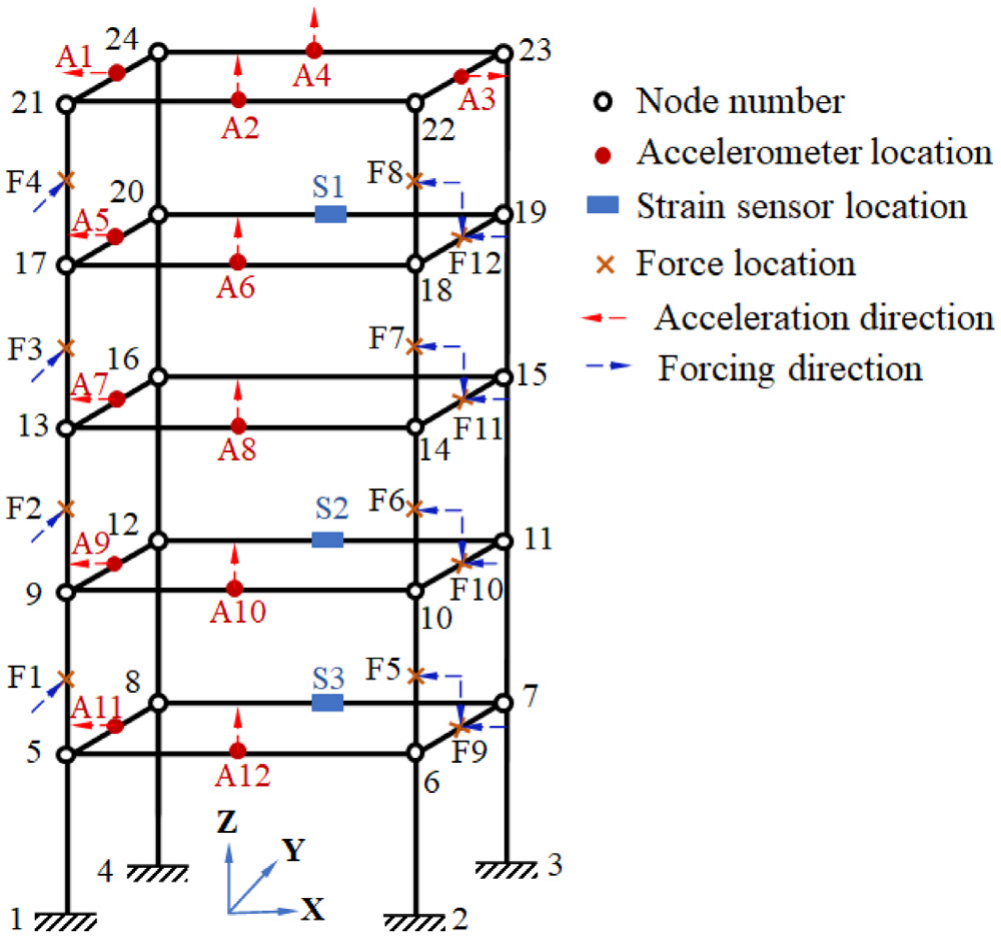
Location details for sensor attachments and force application.

To detail the location and orientation of the sensors and the force exerted on the structure, the locations are numbered and presented in Fig. 10. In this figure, the location of the accelerometers is denoted as A\# (A1-A12).

The orientation of the sensor is further denoted in the figure with a faint arrow line drawn outward from the sensor node denoting its positive axis. The locations for the strain sensors are denoted as S\# (S1–S3). Additionally, the nodes of the structure are numbered (1–24). The application location for force is denoted with F\# (F1–F12) and the corresponding orientation is presented with a faint arrow line drawn inwards towards the force application nodes.

Several experiments are carried out on this test set up and the recorded data are archived. The experimental data contains acceleration time history for the undamaged section as well as the damaged section. The damage location is shown in Fig. 2a, which is at the back side of the longer direction of the third storey. The damage is induced by replacing the undamaged beam with a damaged beam. All the responses are supplied as supplementary data with this article to be used for future research [4].

**Fig. 11:**
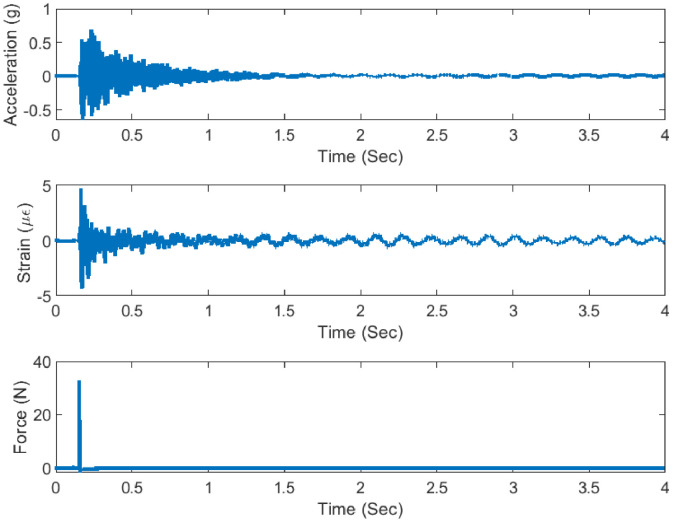
Measured excitation response at A5 location.

The forced vibration test has been conducted for both damaged and undamaged health conditions of the structure. The structure is excited with the input hammer, initial eight force locations (F1–F8) have been hammered in only one direction (8 files) while the remaining four force locations (F9–F12) have been hammered in both horizontal as well as vertical direction leading to four extra experiments (8 files). Altogether, there have been 16 experiments corresponding to 16 files. For each case, there are 16 different force locations and corresponding responses from the acceleration and strain sensors are sampled at a constant sampling rate of 500 Hz (cf. Fig. 11).

The acceleration time history data were also analyzed for modal Parameter estimation using frequency domain decomposition (FDD) Approaches [9,1]. The estimated natural frequencies are presented later in this article [4].

### 2.5 Proposed Numerical Model

It has been mentioned earlier, that for any structural system identification, an assisting model always helps to take decisions, especially for modal parameter estimation. Also, for model based SHM approaches, the requirement of this assisting model becomes imperative. Accordingly, two numerical models have been additionally developed and calibrated to approximately match the real system dynamics.

#### 2.5.1 A High-Fidelity Finite Element Model

A FE model of the experimental setup has been developed with the commercial software package ABAQUS with all the components detailed sufficiently. The objective of this modeling is to perform modal analysis on the numerical replica through which mode matching can be made possible. This model is supplied with the response data.

**Fig. 12:**
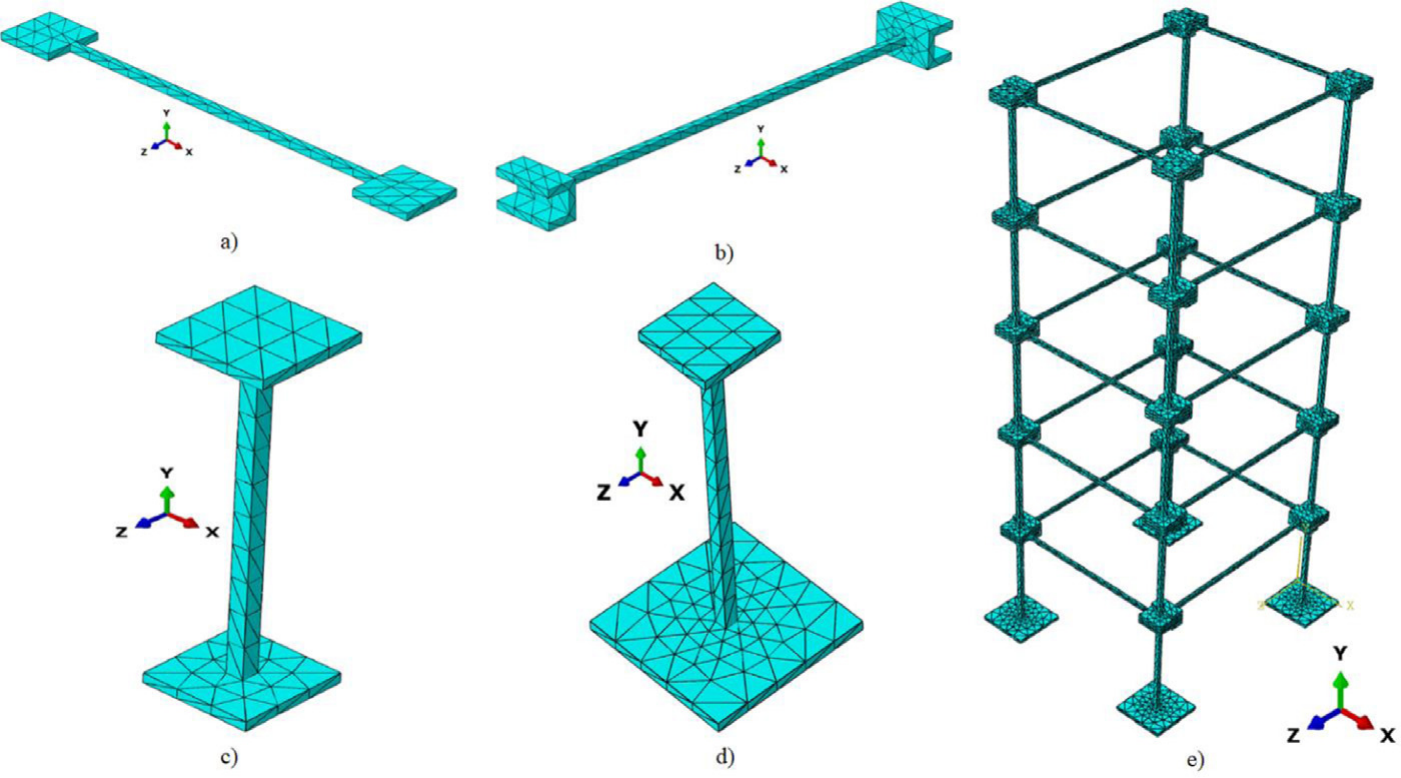
ABAQUS Model with meshing: Component of 3D frame (a) Type A beam, (b) Type B beam, (c) Type X column, (d) Type Y column, and (e) Assemble Model.

Accordingly, the test structure has been modeled using twenty parts each for beams and columns joined together by the tie constraints at their ends, which resist the relative motion between two surfaces (cf. Fig. 12a–d). The base column sets are given fixed boundary conditions with all degrees of freedom constrained. All the parts are sufficiently discretized with 13,838 number of 3D solid elements (10-node quadratic tetrahedron, C3D10 of family 3D stress) and 31,494 numbers of nodes (cf. Fig. 12e). The mesh convergence test has although been performed but not included in this article for the sake of compactness.

**Fig. 13:**
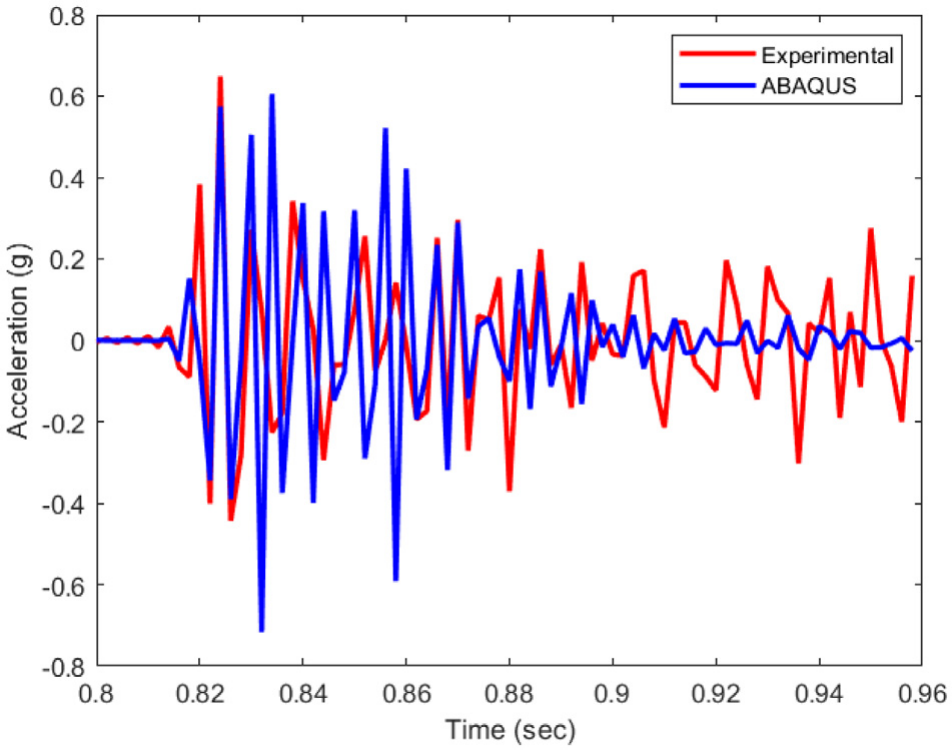
Comparison of acceleration time history.

The experimentally obtained material properties and material nonliterary are further employed in this model. Geometric non-linearity is also assumed for the simulation. Further, transient dynamic analysis has been conducted over structure and comparison of acceleration time histories between real and simulated responses has been shown in Fig. 13. Subsequently, modal analysis has been performed to yield undamped natural frequencies and corresponding mode shapes, which are then compared with the same obtained from the real structure as shown in Table 3. It has been identified that the experimental structure failed to generate sufficient power to excite and exhibit the torsional mode of the frame.

#### 2.5.2 Simplified MATLAB Code

**Fig. 14:**
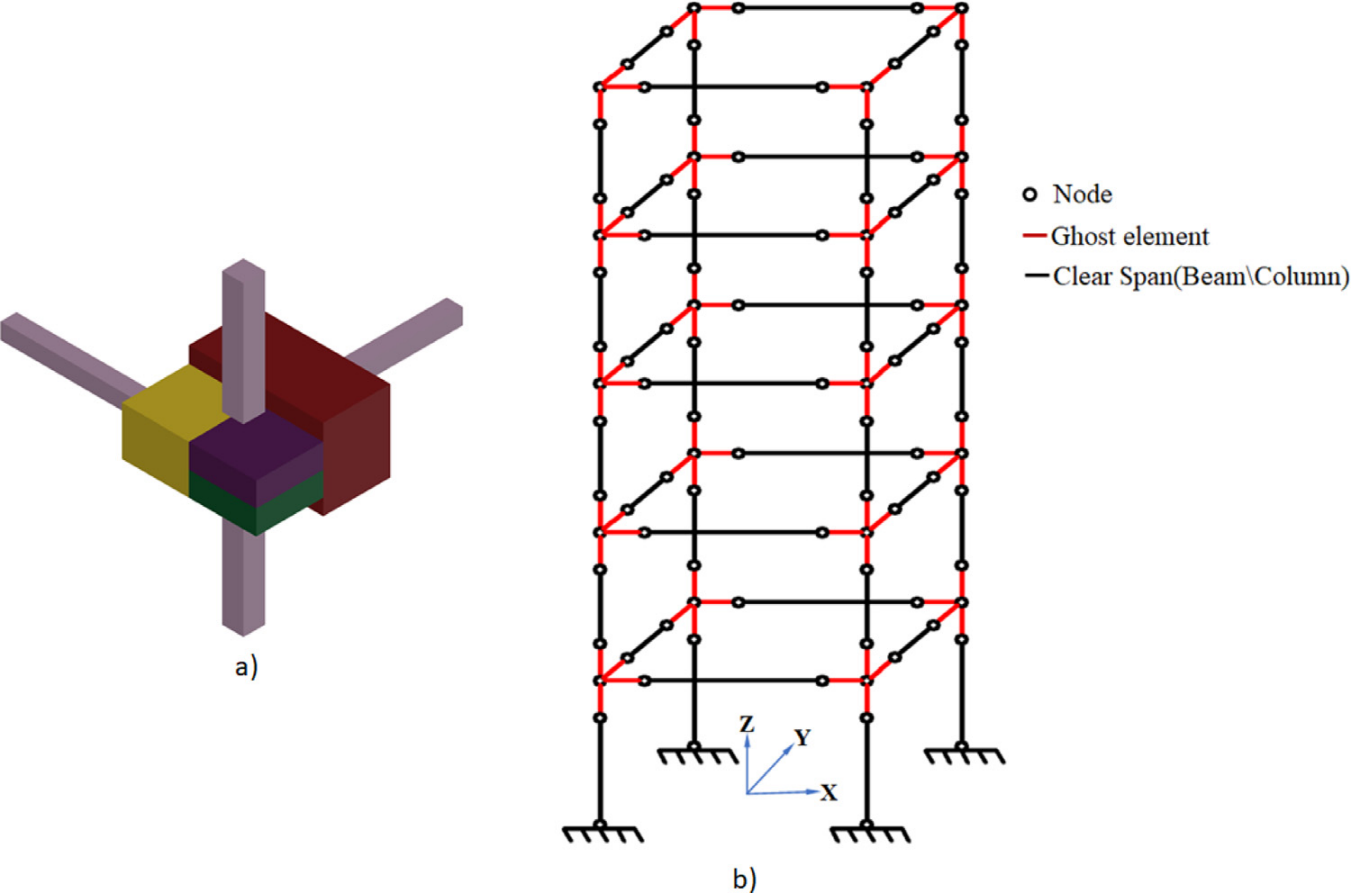
Detail of (a) Magnified 3D Ghost elements (b) Details of Ghost node with element in 3D frame.

Alongside, a simplified MATLAB code has also been generated to numerically mimic the structure with considerably less simulation time. This numerical model is defined with 100 nodes and 116 elements to realize a 576 DOF system. To model the heavy joints, each joint is segmented to distribute its stiffness in terms of thickened beams (in the direction of each member) and a node. Each physical node in the structure is thereby surrounded with four ghost nodes (cf. Fig. 14a). The ghost node and the immediate joint node are further joined with a ghost beam (can also be considered as a thickened beam) having stiffness and mass equivalent to a part of the real joint. To establish the equivalence, the stiffness and mass of the part of the real joint have been replicated with that ghost joint with its elasticity and density factored. Like the previous high-fidelity model, this simplified model is also defined with the material property and nonlinearity definitions for geometry and material. Each of the members (ghost beams or physical members like beams and columns) are defined with two-nodes three-dimensional Euler Bernoulli beam elements each with six DOF. The bottom nodes are again fixed in all directions to realize the bottom fixity connections. The schematic of this numerical model is presented in Fig. 14b. All the details of the modeling, materials, and simulation code are supplied herewith.

Again, modal analysis is performed for this model as well and the modal frequencies so obtained are presented and compared with the same obtained from the real structure in Table 3 and the comparison of frequencies from numerical model with experimental values for damaged Type 1 section, damaged Type 2 sections and damaged Type 3 sections are listed in Table 4, Table 5 and Table 6, respectively.

**Table 6:** Comparison of natural frequencies (Hz) for type 3 damage.

		Deviation (%)
Experimental	ABAQUS	∈ae
5.55	6.205	11.807
6.775	6.567	3.077
-	7.867	-
20.57	21.320	3.646
23.38	22.254	4.816
-	24.146	-

Where ∈ represents error in the quantity while the subscript *a, m*, or *e* represents the association of the error to Abaqus, MATLAB or real experiment.

## Ethics Statements

All the individuals involved have been properly credited.

## CRediT authorship contribution statement

**Md Armanul Hoda:** Methodology, Data curation, Software, Investigation, Validation, Writing – original draft. **Eshwar Kuncham:** Supervision, Visualization, Software, Investigation. **Subhamoy Sen:** Conceptualization, Methodology, Supervision, Writing – review & editing.

## Declaration of Competing Interest

The authors declare that they have no known competing financial interests or personal relationships that could have influenced the work reported in this paper.

## Data Availability

Time history response and numerical models of a 3D shear frame (Original Data) (Mendeley Data).
